# Mortality benefits of reduction fine particulate matter in Vietnam, 2019

**DOI:** 10.3389/fpubh.2022.1056370

**Published:** 2022-11-18

**Authors:** Nguyen Thi Trang Nhung, Vu Tri Duc, Vo Duc Ngoc, Tran Minh Dien, Le Tu Hoang, Tran Thi Thuy Ha, Pham Minh Khue, Ngo Xuan Truong, Nguyen Thi Nhat Thanh, Edward Jegasothy, Guy B. Marks, Geoffrey Morgan

**Affiliations:** ^1^Department of Biostatistics, Faculty of Fundamental Sciences, Hanoi University of Public Health, Hanoi, Vietnam; ^2^Vietnam National Children's Hospital, The Training and Research Institute for Child Health, Hanoi, Vietnam; ^3^Faculty of Public Health, Haiphong University of Medicine and Pharmacy, Haiphong, Vietnam; ^4^University of Engineering and Technology, Vietnam National University, Hanoi, Vietnam; ^5^Faculty of Medicine and Health, Sydney School of Public Health, University Centre for Rural Health, University of Sydney, Sydney, NSW, Australia; ^6^Centre for Air Pollution, Energy and Health Research, University of New South Wales, Sydney, NSW, Australia

**Keywords:** air pollution, PM_2.5_, Vietnam, mortality burden, health benefits

## Abstract

**Introduction and objectives:**

Studies assessing the health benefits of air pollution reduction in Vietnam are scarce. This study quantified the annual mortality burden due to PM_2.5_ pollution in Vietnam above the World Health Organization recommendation for community health (AQG: 5 μg/m^3^) and the proposed National Technical Regulation on Ambient Air Quality (*proposed QCVN:* 15 μg/m^3^).

**Methodology:**

This study applied a health impact assessment methodology with the hazard risk function for non-communicable diseases (NCDs) and lower respiratory infections (LRIs) in the Global Exposure Mortality Model (GEMM) to calculate attributable deaths, Years of Life lost, and Loss of Life expectancy at birth due to air pollution in the Vietnamese population above 25 years of age in 11 provinces. We obtained annual average PM_2.5_ concentrations for Vietnam in 2019 at a 3x3 km grid modeled using Mixed Linear regression and multi-data sources. Population and baseline mortality data were obtained from administrative data system in Vietnam. We reported the findings at both the provincial and smaller district levels.

**Results:**

Annual PM_2.5_ concentrations in all studied provinces exceeded both the AQG and the *proposed QCVN*. The maximum annual number of attributable deaths in the studied provinces if they had complied with WHO air quality guidelines was in Ha Noi City, with 5,090 (95%CI: 4,253–5,888) attributable deaths. At the district level, the highest annual rate of attributable deaths if the WHO recommendation for community health had been met was 104.6 (95%CI: 87.0–121.5) attributable deaths per 100,000 population in Ly Nhan (Ha Nam province).

**Conclusion:**

A much larger number of premature deaths in Vietnam could potentially be avoided by lowering the recommended air quality standard. These results highlight the need for effective clean air action plans by local authorities to reduce air pollution and improve community health.

## Introduction

Fine particulate matter (denoted as PM_2.5_) concentrations have declined globally for the last decade. For example, the annual mean PM_2.5_ in the United States was reduced by approximately 41% from 2000 to 2020 (1). In Europe, the decrease in PM_2.5_ concentrations across countries was 29% from 2005 to 2019 (2). In China, a decline in annual PM_2.5_ concentration was observed, ranging from 22.2 to 56.6% during 2015–2020 (3). These improvements in outdoor air quality throughout the world are the result of affordable clean air action. For example, the “Air Pollution Prevention and Control Action Plan,” first implemented in China in 2013, contributed to 15% reduction in PM_2.5_ in the Pearl River Delta (4). In other words, evidence quantifying the health benefits of reducing exposure to PM_2.5_ is urgently needed to support further clean air policy development and implementation, particularly in high-exposure countries such as Vietnam.

Though air quality in Vietnam has improved in recent years, it is still poor by international comparisons. Annual mean PM_2.5_ concentrations in Vietnamese provinces ranged from 9–41 μg/m^3^ in 2019 to 8–36 μg/m^3^ in 2020 (5). However, the annual PM_2.5_ concentration in 10 out of 63 provinces in Vietnam in 2020 exceeded the National Technical Regulation on Ambient Air Quality- QCVN 05:2013 (set at 25 μg/m^3^), and no provinces reached the new World Health Organization's air quality guidelines (AQG, set at 5 μg/m^3^) (5, 6). Provinces in the Red River Delta such as Hung Yen (32.7 μg/m^3^) and Bac Ninh (33.0 μg/m^3^) experienced the poorest air quality, rather than the major cities such as Ha Noi and Ho Chi Minh City (5).

Exposure to the high concentration of PM_2.5_ can cause many diseases including cardiovascular, respiratory diseases, diabetes, lung cancer (7), and contribute to premature deaths (8–10). The evidence also indicates no threshold of air pollutant concentration for community health. The effect of PM_2.5_ remained even where the exposure level is low such as in the European Union region and Australia (7, 11, 12). Therefore, in September 2021, the World Health Organization (WHO) updated its 2015 recommendation in which it set the annual mean concentration of PM_2.5_ at 5 μg/m^3^ (6). Though this guideline is not a regulation, it can drive the air quality policy worldwide.

In recent years, the Vietnamese government has attempted to address poor air quality through a series of actions. In 2016, the Vietnamese Prime Minister issued the National Action Plan on air quality management by 2020 including a vision for 2025 (Decision No 985a/QÐ-TTG) which targeted improved air quality management by controlling emissions and increasing ambient air quality monitoring (13). Directive No 03/CT-TTg on enhancing air quality management was signed by the Prime Minister on 18 January 2021 (14). This latter document also called for the assessment of the adverse health impacts of air pollution on community health (14). Decision No 1973/QÐ-TTg states that Vietnam will take actions to improve air quality management *via* controlling emissions and monitoring ambient air pollutants and also conduct research to provide information *via* early warning systems that predict air quality in each province (15). These documents emphasize the importance of assessing the health impacts of air pollution to evaluate the implemented policy and associated interventions in local areas (13–15). The Official Dispatch No 3051/BTNMT-TCMT on the technical guidelines of building air management plan at the provincial level recommends the use of AirQ+ software (a software developed by the World Health Organization–WHO) to assess the health impacts of air pollution (16). Ha Noi, in particular, issued Directive No 15/CT-UBND on entirely replacing and eliminating the use of the beehive coal fuel to reduce the adverse impacts of air pollution in the city (17). Also, in 2021, the Ministry of Natural Resources and Environment issued a draft of the National Technical Regulation on Ambient Air Quality (called proposed QCVN in this study) (18) in order to establish a policy to improve ambient air quality in Vietnam. This study quantifies the mortality impact of air pollution in Vietnam in response to the call for such policies by the Vietnamese government.

Evidence of the health impacts of ambient air pollution in Vietnam is scarce, and mainly in Ha Noi and Ho Chi Minh City. Two studies showed an association between air pollutants, including PM_2.5_, and hospital admissions amongst Hanoi children (19, 20). Another study demonstrated the relationship between ambient air pollutants and hospital admissions for cardiovascular and respiratory diseases in adults in Hanoi, Quang Ninh, and Phu Tho (21) and a study in Ho Chi Minh City showed the association between hospitalization due to acute lower respiratory infection and PM_2.5_ (22). The first Vietnamese health impact assessment study of exposure to PM_2.5_ was conducted in Ha Noi in 2017 (23). The findings from this study showed the life expectancy at birth of the Hanoi population due to exposure to PM_2.5_ had decreased by 1.8 years in 2017 (23). Research in Ho Chi Minh City also found that 1,136 premature deaths were attributed to PM_2.5_ in 2017 (24). Such findings highlight the large burden of diseases due to PM_2.5_ exposure in Vietnam.

This study aimed to quantify the health burden due to ambient PM_2.5_ concentrations above the World Health Organization guideline and the proposed National Technical Regulation on Ambient Air Quality in several provinces of Vietnam, including Bac Ninh, Hung Yen, Hai Duong, Ha Noi, Thai Binh, Hai Phong, Ninh Binh, Ha Nam, Ho Chi Minh, Quang Ninh, and Dien Bien. The burden of mortality is expressed as attributable deaths (AD) (number and rate per 100,000 population), Years of life lost (YLL) (number and rate per 100,000 population), and Loss of Life Expectancy (LLE) (in years) for these provinces in Vietnam. Moreover, we also calculated the attributable deaths (rate per 100,000 population) using the AirQ+ software to compare our results with the estimate according to the technical guidelines of the Official Dispatch No 3051/BTNMT-TCMT of the Ministry of Natural Resources and Environmental (16). This study not only provides scientific data on the health impact of air pollution in Vietnam but also responds to the Vietnamese government's call for evidence to establish air quality control that will improve health.

## Materials and methods

### Study areas

We conducted a health impact assessment of ambient PM_2.5_ concentrations in 11 Vietnamese provinces, including 10 provinces in the northern region (Ha Noi, Bac Ninh, Hung Yen, Ha Nam, Hai Duong, Thai Binh, Hai Phong, Ninh Binh, Quang Ninh, Dien Bien) and Ho Chi Minh City in the south of Vietnam. The total 2019 population of Vietnam was 96,208,984 people and our study region included an exposed population of 18,342,836 (19%) inhabitants (25) covering the most polluted provinces (5) and also the two megacities of Hanoi (the capital) and Ho Chi Minh City.

### PM_2.5_ exposure

We assessed exposure using a previously published study (23). Daily PM_2.5_ maps for Vietnam at 3x3 km resolution were estimated by Mixed Effect Models developed on a 9-year dataset (2012-2020) of PM_2.5_ measured at ground stations, combined with satellite Aerosol Optical Depth (AOD), meteorological conditions (i.e., humidity, planetary boundary layer height) and land use (i.e., normalized difference vegetation index (NDVI), road density) (26). The detailed method, data source, and model validation for estimating daily PM_2.5_ maps is described in our previous study (23). The daily mean PM_2.5_ maps were validated by comparing them with ground-based PM_2.5_ measurements, with a Pearson coefficient (r) of 0.87 and a Root Mean Square Error (RMSE) of 11.76 μg/m3. The daily maps were then aggregated into monthly maps and annual maps. The annual average PM_2.5_ map in 2019 had high agreement with ground PM_2.5_ measurements (r = 0.84, RMSE = 5.07 μg/m3). The annual PM_2.5_ mean concentration data in 2019 was calculated for each district of the selected provinces for the health impact assessment process.

### Mortality data and exposure population

Death cases including age, address (district and province), gender, and specific causes of death were obtained from the Vietnam A6 registration. A6 document is a death registration at the commune health, the lowest level of the health sector in Vietnam—and data on death cases of permanent inhabitants was collected by community health staff. This document covers approximately 89% of the total deaths in the commune (27). The report also confirmed that data on death due to injury is robust (27) and so we were able to exclude injury-related deaths from our analysis.

The number of inhabitants in each district by age group and gender were extracted from the Population and Household census in 2019 by the General Statistics Office of Vietnam (hereafter called GSO) (25, 28). This census obtained information on the population including fertility, gender, migration, urbanization, and population aging in Vietnam.

### Health impact assessment estimation

We applied the Global Exposure Mortality Model (GEMM) risk function to estimate the attributable deaths due to ambient PM_2.5_ pollution (29) for each province. Burnett et al. developed GEMM by pooling hazard ratios from 41 cohorts (29), including the Chinese cohort where the ambient PM_2.5_ concentration and emission sources are more similar to Vietnam compared to European and North American studies.

In each province, we estimated attributable deaths (AD), years of life lost (YLL) and loss of life expectancy (LLE) due to air pollution for populations above 25 years of age in each province separately. The AD for each 5-year age subgroup in a district is estimated from applying the hazard ratio to specific age groups and PM_2.5_ concentration using the GEMM risk function (29), the death rate for each age group in a district, and the population at that age group for the corresponding district. We applied the estimate for non-communicable diseases (NCDs) and lower respiratory infections (LRIs) in GEMM (denoted as GEMM NCD+LRI). We set the counterfactual concentration as defined by 1) the WHO air quality guidelines (AQG: 5 μg/m^3^) and 2) the proposed QCVN (15 μg/m^3^). YLL is calculated by multiplying the attributable deaths in each age group by the life expectancy in the corresponding age group. This life expectancy is calculated by applying the life-table method with the baseline death rate per 5-year age group in 2019. Thirdly, the LLE in days was calculated by subtracting the life expectancy without the contribution of PM_2.5_ to the PM_2.5_ attributed life expectancy. For each province and district, LLE was expressed in years by dividing the LLE in days by 365.25. The rates of deaths and YLL for each district per 100,000 inhabitants were calculated by dividing the number of the corresponding indicators by the population size. We present the findings at the provincial and district levels.

The other analysis was conducted in R version 4.0.3 (R Core Team Vienna, R Foundation for Statistical Computing, Vienna, Austria) with the iomlifetR package (30), apart from the AirQ+ comparison calculaltions.

### Comparison analysis

For comparison, we also estimated the attributable deaths (rate per 100,000 population) by province using the AirQ+ software and selecting the long-term impact assessment option for total natural causes. By default this option utilized the log-linear model with the relative risks retrieved from the literature and meta-analysis of Hoek et al. (31). Since this model only applies to adults 30 years of age or older, we only calculated attributable deaths for this population *via* AirQ+.

## Results

Population and mortality data for people above 25 years old in 11 provinces in 2019 are provided in Table 1. Ha Noi and Ho Chi Minh were the two most populous areas, with the population over 25 years old reaching approximately 5 to 6 million inhabitants. Crude death rates by province ranged from 4.1 deaths per 1,000 population in Hung Yen to 8.3 deaths per 1,000 population in Thai Binh.

**Table 1:** Annual means of the PM_2.5_ concentration (in μg/m^3^), population and death rates per 1,000 population above age 25 years in 11 study region provinces, Vietnam, 2019.

**Province's name**	**PM_2.5_ annual mean concentration (μg/m^3^)**	**Population above age 25**	**Death rates above aged 25+ years (per 1,000 population)**
Ha Noi	40.8	4,889,379	4.6
Bac Ninh	37.8	812,711	5.4
Hung Yen	35.2	789,408	4.1
Ha Nam	31.5	543,823	7.9
Hai Duong	31.2	1,222,568	4.3
Thai Binh	27.1	1,237,051	8.3
Hai Phong	25.8	1,311,556	7.4
Ninh Binh	23.5	622,278	7.8
Ho Chi Minh	20.9	5,795,749	4.8
Quang Ninh	18.9	825,247	5.8
Dien Bien	15.8	293,066	6.3

**Figure 1:**
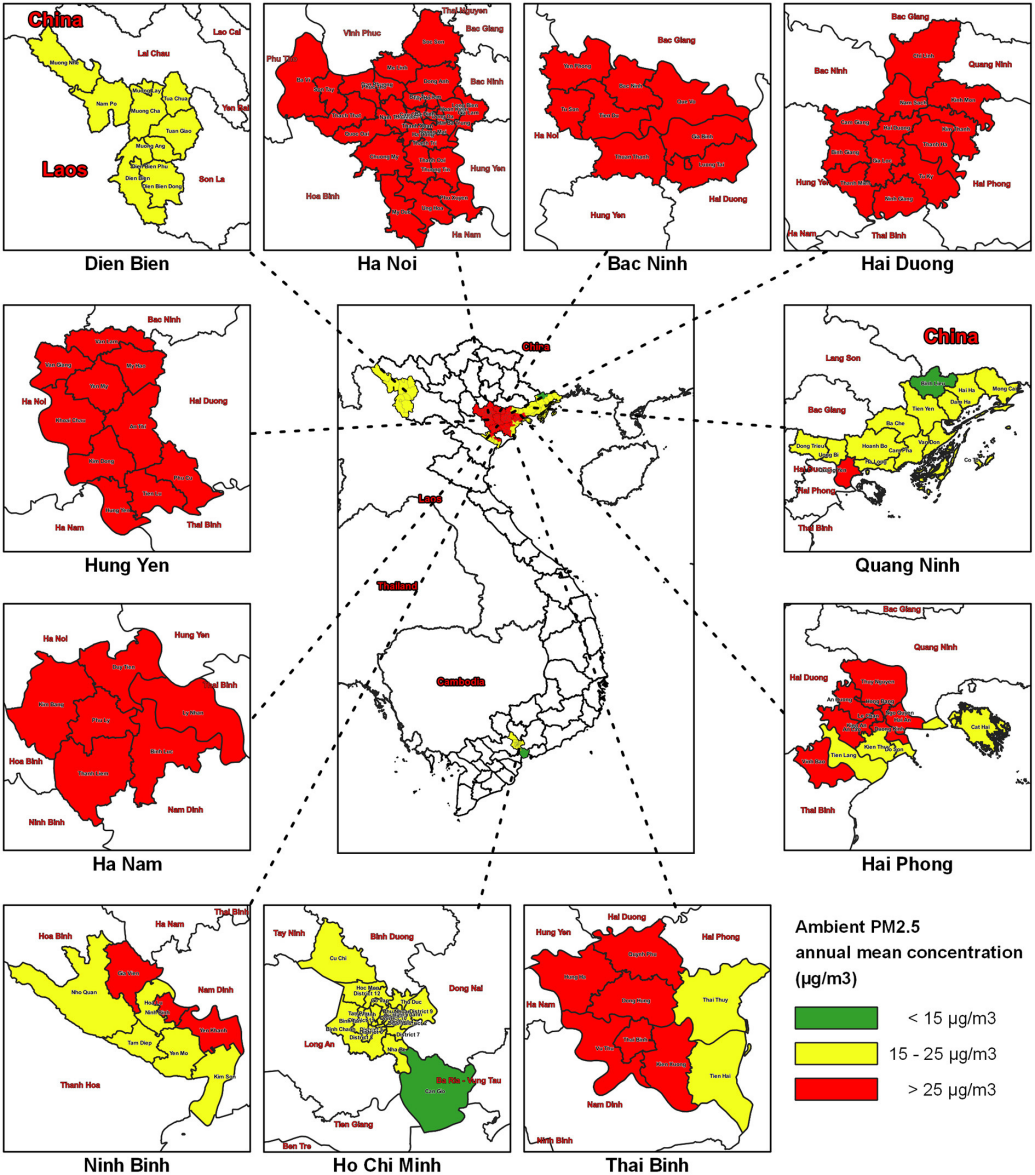
PM_2.5_ concentrations by districts in the 11 study region provinces of Vietnam, 2019.

The annual PM_2.5_ concentration ranged from 15.8 μg/m3 in Dien Bien to 40.8 μg/m3 in Ha Noi (Table 1, Figure 1). All annual mean concentrations in each of the 11 provinces exceeded the WHO air quality guidelines and the proposed QCVN. Notably, Bac Ninh, Hung Yen and Ha Nam, adjacent to Hanoi, experienced highly polluted levels. The annual mean of PM_2.5_ concentration in Ha Noi was 2.72 times higher than the proposed QCVN (Table 1, Figure 1).

At the district level, the highest PM_2.5_ concentration was observed in all districts of Bac Ninh, Hai Duong, Hung Yen, Ha Nam province, and the central districts of the capital Ha Noi (Figure 1).

Compliance with the WHO air pollution guideline could potentially avoid 5,090 (95%CI: 4,253–5,888) annual premature deaths in Hanoi from exposure to PM_2.5_ (Table 2) and 4,076 (95% CI: 3,377–4,754) premature deaths in Ho Chi Minh City. The highest PM_2.5_ attributable death rates were Ha Nam, Thai Binh, and Hai Phong, with 95.1 (95% CI: 79.1–110.4), 93.4 (95% CI: 77.6–108.7), and 81.9 (95% CI: 68.0–95.3) deaths per 100,000 population, respectively.

**Table 2:** Avoidable deaths, years of life lost and loss of life expectancy (expressed in number and rate per 100,000 population) and confidence interval (95% CI) when compliance with The World Health Organization's Air Quality Guidelines in Vietnam study region provinces, 2019.

**Province's name**	**Avoidable deaths**		**Years of life lost**		**Loss of life expectancy (years) (95%CI)**
	**Number (95%CI)**	**Rate per 100,000 population (95%CI)**	**Number (95%CI)**	**Rate per 100,000 population (95%CI)**	
Ha Noi	5,090 (4,253–5,888)	63.2 (52.8–73.1)	152,828.6 (124,123.8–181,963.7)	1,897.6 (1,541.2–2,259.4)	4.9 (3.9–5.9)
Bac Ninh	946 (789–1,096)	69.1 (57.7–80.0)	23,779.8 (19,353.7–28,252.8)	1,737.2 (1,413.9–2,064.0)	3.8 (3.1–4.6)
Hung Yen	658 (548–763)	52.5 (43.7–60.9)	23,940.0 (19,392.6–28,573.7)	1,911.0 (1,548.0–2,280.9)	5.7 (4.6–6.8)
Ha Nam	811 (675–942)	95.1 (79.1–110.4)	19,520.0 (15,881.1–23,196.9)	2,288.9 (1,862.2–2,720.1)	3.4 (2.8–4.1)
Hai Duong	968 (805–1,124)	51.1 (42.5–59.4)	32,989.8 (26,753.7–39,327.5)	1,743.4 (1,413.9–2,078.3)	4.8 (3.9–5.8)
Thai Binh	1,738 (1,443–2,022)	93.4 (77.6–108.7)	39,772.4 (32,368.7–47,244.1)	2,137.8 (1,739.8–2,539.4)	2.9 (2.4–3.5)
Hai Phong	1,661 (1,380–1,933)	81.9 (68.0–95.3)	38,360.9 (31,294.0–45,461.7)	1,891.7 (1,543.2–2,241.8)	2.8 (2.2–3.3)
Ninh Binh	758 (628–883)	77.1 (64.0–89.8)	18,422.6 (15,029.6–21,830.2)	1,875.1 (1,529.8–2,221.9)	2.7 (2.2–3.2)
Ho Chi Minh	4,076 (3,377–4,754)	45.3 (37.5–52.9)	88,710.3 (72,525.0–104,897.0)	986.4 (806.5–1,166.4)	1.9 (1.6–2.3)
Quang Ninh	687 (569–801)	52.0 (43.1–60.7)	18,436.3 (15,042.3–21,844.0)	1,396.3 (1,139.3–1,654.4)	2.5 (2.1–3.0)
Dien Bien	222 (183–259)	37.0 (30.6–43.3)	6,437.5 (5,258.7–7,617.9)	1,075.0 (878.1–1,272.1)	2.2 (1.8–2.6)

Our study also observed great variability in attributable deaths at district levels (Supplementary Table S1). The attributable death rates range from 22.3 deaths per 100,000 population in Muong Nhe district (Dien Bien) to 104.6 deaths in Ly Nhan (Ha Nam province). In Ha Noi, the capital of Vietnam, districts with the greatest attributable death rate are located in the central districts (Hoan Kiem, Ba Dinh, Hai Ba Trung, Dong Da, and Tay Ho) of the province.

**Figure 2:**
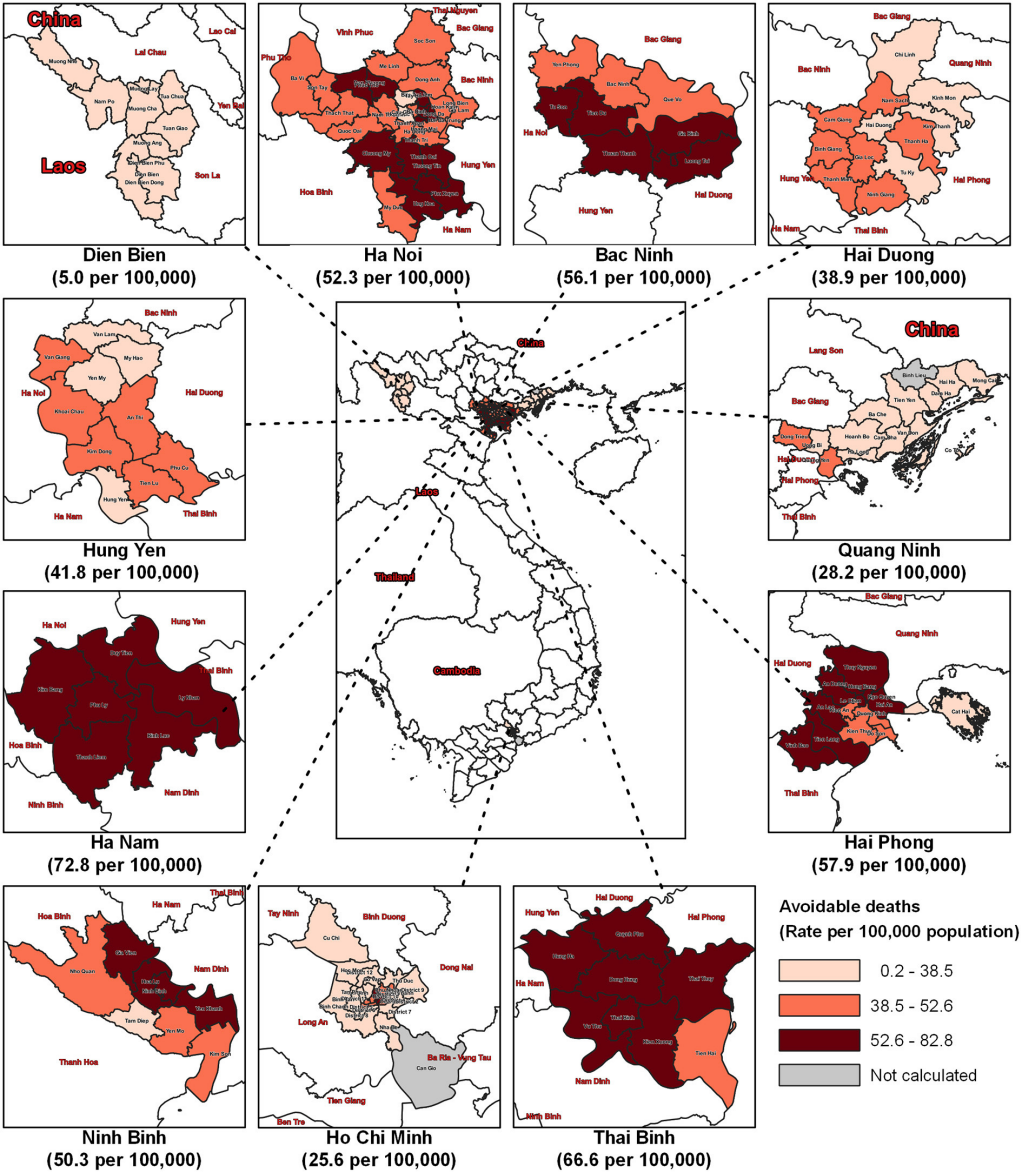
Attributable deaths (expressed in rate per 100,000 population) by districts in 11 provinces in Vietnam due to PM_2.5_ concentrations above the proposed QCVN (15 μg/m^3^) 2019. The “Not calculated” areas had the PM_2.5_ concentration lower than the proposed QCVN (15 μg/m^3^).

Figure 2 illustrates the number and the rate (per 100,000 population) of attribute deaths due to PM_2.5_ concentrations above the proposed QCVN. The rate of attributable deaths due to annual ambient PM_2.5_ in the selected provinces above the proposed QCVN was 52.3 deaths per 100,000 population in Ha Noi and 66.6 per 100,000 population in Thai Binh.

## Discussion

In this study, we estimated 37.0 to 95.1 deaths per 100,000 population are attributable to PM_2.5_ pollution in 2019 above the WHO air quality guidelines for annual average PM_2.5_ in the selected provinces. These numbers were generally higher than the PM_2.5_ attributable mortality rate in Vietnam (44.8 per 100,000 population) according to the calculation of the Global Burden of Disease (GBD) study (32). The discrepancy will be influenced by the difference in resolution of the PM_2.5_ concentration maps we used of 3 x 3 km that is smaller (higher spatial resolution) compared to the air pollution maps used in the GBD. Nonetheless, both these analyses present a high rate of attributable mortality due to exposure to PM_2.5_ in Vietnam and highlight the urgency to effectively implement the National Action Plan for air quality management in Vietnam.

To address this issue, the Prime Minister of Vietnam stated 6 objectives in Decision No 1973/QÐ-TTg in 2021, including: 1) Complete the structure, policies, and constitutions regarding air quality management; 2) Prevent and reduce emission; 3) Complete the financial structure and diversify the investment for air quality management; 4) International cooperation and scientific research regarding technology and air quality management; 5) Monitor and evaluate the compliance of air quality management; 6) Communicate, educate, and improve the capacity and acknowledgment regarding air quality management (15). Implementing these objectives is an interdisciplinary task and requires the involvement of the entire Vietnamese administrative structures (the details of the assignments were described in Decision No 1973/QÐ-TTg and the Law 72/2020/QH14 on Environmental Protection) (15, 33). Our study findings demonstrate the health benefits which Vietnam can obtain from effectively implementing air pollution control measures. Parallel with the above strategies, the Vietnam government has planned to reduce QCVN from 25 μg/m^3^ to 15 μg/m^3^. However, the proposed QCVN and a time frame for achieving this standard have not yet been officially issued.

The Official Dispatch No. 3051/BTNMT-TCMT suggests using the AirQ+ software developed by the WHO (16). In this study, the death rates for provinces estimated by AirQ+ were higher than the findings using GEMM, though the exposure population is marginally smaller (Table 3). The likely reason for these differences is that the population attributable fraction estimated by GEMM compared to that estimated using the Hoek et al. (31) risk function at lower PM_2.5_ concentrations, but the opposite situation occurs when the PM_2.5_ concentration is high (34). Therefore, using the log-linear model in AiQ+ for Vietnam might overestimate the actual burden of mortality due to the high ambient PM_2.5_ concentrations.

**Table 3:** Attributable deaths (rate per 100,000) between the calculation by Log–linear model in AirQ+ and the calculation by GEMM for each province.

**Province's name**	**Attributable deaths (Rate per 100,000 population) by Log–linear model in AirQ + (95%CI)**	**Attributable deaths (Rate per 100,000 population) by GEMM(95%CI)**
Ha Noi	103.7	63.2
Bac Ninh	118.8	69.1
Hung Yen	79.3	52.5
Ha Nam	132.8	95.1
Hai Duong	70.5	51.1
Thai Binh	115.2	93.4
Hai Phong	103.7	81.9
Ninh Binh	95.4	77.1
Ho Chi Minh	53	45.3
Quang Ninh	59.7	52.5
Dien Bien	46.6	37.0

The attributable mortality rates in the central northern provinces (such as Thai Binh or Ha Nam and Hai Duong) were high and this is likely due to the industrial areas located in these provinces (35). A greater number of thermal power stations, cement manufacturer factories, and factories with older technology are located in the northern region, especially in Hai Duong and Thai Binh (35). Moreover, Bac Ninh includes many villages with traditional industries that lack pollution control systems (35). Our study indicates that the substantial health burden due to air pollution in the Bac Ninh province could be reduced by implementing effective interventions to manage the air quality in these polluted areas.

The National Action Plan on air quality management for 2021–2025 in Vietnam is in its initial stages and authorities should be aware of the range of air pollutants that need to be reduced to decrease the health burden (15). In this study, we primarily focus on the mortality burden due to a reduction in PM_2.5_. A study in China found an inverse association between PM_2.5_ and ground-level Ozone (O_3_) concentration (36). According to the authors reducing the Nitrogen Oxide (NO_x_) and Volatile Organic Compounds (VOCs) components in PM_2.5_ could potentially increase the photochemical formation of O_3_, thus resulting in the higher concentration of O_3_. Ground-level ozone has been found to have an impact on hospital admissions in Vietnam (37). Therefore, future interventions should consider the proportion of air pollutants in the mixture and their interrelation before implementing interventions to reduce any specific pollutant.

In this study, we applied the GEMM risk function to estimate the attributable deaths due to ambient PM_2.5_. This risk function has been used in numerous studies to assess not only the health impact of air pollution, but also the economic health benefits of an intervention. For example, Haikun Wang (2020) used GEMM to estimate the health benefits of control programs relating to on-road transportation in China during the period of 1998 to 2015 (38). Another study used GEMM to assess the health and economic impact of China's air pollution shift from 2013 to 2018, as well as to suggest implications for air pollution control policies during this time period (36). This approach is more suitable for countries and regions with severe pollution. The estimation is similar to the GBD approach. In addition, the Environmental Benefits Mapping and Analysis Program—Community Edition (BenMAP-CE) by the United States Environmental Protection Agency integrated this approach into the software; therefore so it can be easily applied (39). We recommend that Vietnam use this approach to estimate both the health and economic benefits of interventions to reduce air pollution.

Our study has several limitations. While we were unable to collect data from all the areas in Vietnam we obtained data for the most polluted provinces, including both the north and south of Vietnam. The mortality data used in our analysis is estimated to include around 89% of all deaths based on a study in Quang Ninh and Thai Nguyen provinces (27). Although we performed the quality assurance process on the mortality data (details of the procedure were described elsewhere (27)) the attributable mortality due to air pollution we estimated is likely to be an underestimate due to the <100% covered of the mortality data we used. The air quality data we used was modeled using data from a limited number of fixed site air pollution monitoring stations in Vietnam and this could introduce error in our estimated PM_2.5_ exposures. Our study applied a hazard ratio obtained from the analysis of 41 cohorts, including China (29). While this hazard ratio is estimated from a number of large cohort studies there may be some differences when generalizing to different populations.

## Conclusion

Our study found that there is a substantial burden of mortality due to air pollution in Vietnam above the air quality guidelines by the WHO and the proposed Vietnam QCVN. These findings highlight the health benefits of reducing air pollution in Vietnam including actions by local provincial and district authorities to implement policies to improve air quality.

## Data availability statement

The original contributions presented in the study are included in the article/Supplementary material, further inquiries can be directed to the corresponding author.

## Ethics statement

The studies involving human participants were reviewed and approved by the Ethics Committee of the Hanoi University of Public Health (Ref. No. 020-265/DD-YTCC). Written informed consent for participation was not required for this study in accordance with the National Legislation and the Institutional Requirements.

## Author contributions

NTTN, GBM, and GM conceived, designed the study, and acquired data. NTNT and NXT conducted statistical analysis for PM_2.5_ concentration. NTTN, VTD, VDN, and LTH conducted statistical analyses for health impact assessment. NTTN and VTD drafted the manuscript. All authors provide statistical support, interpretation, professional support, and made several critical revisions to the manuscript. All authors read and approved the final manuscript.

## Funding

This study received funding from Vietnam National Foundation for Science and Technology Development (NAFOSTED) under grant number 105.08–2019.331. The funder was not involved in the study design, collection, analysis, interpretation of data, the writing of this article, or the decision to submit it for publication.

## Conflict of interest

The authors declare that the research was conducted in the absence of any commercial or financial relationships that could be construed as a potential conflict of interest.

## Publisher's note

All claims expressed in this article are solely those of the authors and do not necessarily represent those of their affiliated organizations, or those of the publisher, the editors and the reviewers. Any product that may be evaluated in this article, or claim that may be made by its manufacturer, is not guaranteed or endorsed by the publisher.
