# Supplementary material for: Mortality benefits of reduction fine particulate matter in Vietnam, 2019
Source: Front Public Health. 2022 Nov 18;10:1056370. doi: 10.3389/fpubh.2022.1056370 (PMC9718030; doi:10.3389/fpubh.2022.1056370)
Supplement: Supplementary file 1 [file Data_Sheet_1.pdf]

Supplementary Material

Supplementary Table 1

Attributable deaths (number and rate per 100,000 population) and confidence interval (95% CI) by provinces and districts when Vietnam compliant with Ambient Quality Guideline (AQG) by the World Health Organization, Vietnam, 2019

Provinces and districts	Avoidable deaths		Years of life lost		Loss of life expectancy (years) (95%CI)
	Number (95%CI)	Rate per 100,000 population (95%CI)	Number (95%CI)	Rate per 100,000 population (95%CI)	
Ha Noi*	***5,090 (4,253 - 5,888)***	***63.2 (52.8 - 73.1)***	***152,828.6 (124,123.8 - 181,963.7)***	***1,897.6 (1,541.2 - 2,259.4)***	***4.9 (3.9 - 5.9)***
Nam Tu Liem	116 (97 - 133)	43.8 (36.7 - 50.5)	3,819.3 (3,107.9 - 4,539.7)	1,445.4 (1,176.1 - 1,718.0)	5.5 (4.4 - 6.6)
Thanh Xuan	184 (154 - 212)	62.6 (52.4 - 72.2)	5,778.1 (4,695.3 - 6,876.7)	1,968.5 (1,599.6 - 2,342.8)	5.3 (4.3 - 6.4)
Thanh Oai	153 (128 - 177)	72.5 (60.6 - 83.7)	4,539.4 (3,681.4 - 5,413.0)	2,151.1 (1,744.5 - 2,565.0)	5.2 (4.2 - 6.3)
Ha Dong	204 (171 - 235)	51.2 (42.9 - 59.1)	6,541.3 (5,320.8 - 7,777.3)	1,644.1 (1,337.4 - 1,954.8)	5.2 (4.2 - 6.3)
Cau Giay	162 (136 - 187)	55.5 (46.4 - 64.1)	5,068.8 (4,119.0 - 6,032.4)	1,732.7 (1,408.0 - 2,062.1)	5.2 (4.2 - 6.3)
Bac Tu Liem	143 (120 - 166)	42.8 (35.8 - 49.4)	4,644.5 (3,779.9 - 5,519.2)	1,386.0 (1,128.0 - 1,647.0)	5.1 (4.1 - 6.2)
Hoang Mai	270 (226 - 312)	53.3 (44.6 - 61.5)	8,557.2 (6,959.7 - 10,175.2)	1,690.0 (1,374.5 - 2,009.5)	5.1 (4.1 - 6.1)
Thuong Tin	185 (155 - 214)	72.7 (60.8 - 84.0)	5,484.3 (4,449.3 - 6,537.2)	2,153.2 (1,746.9 - 2,566.6)	5.1 (4.1 - 6.1)

Thanh Tri	149 (125 - 172)	54.1 (45.2 - 62.5)	4,725.4 (3,843.2 - 5,619.1)	1,713.7 (1,393.7 - 2,037.8)	5.1 (4.1 - 6.1)
Hoai Duc	155 (129 - 179)	58.8 (49.2 - 67.9)	4,714.1 (3,828.9 - 5,612.7)	1,792.6 (1,456.0 - 2,134.3)	5.1 (4.1 - 6.1)
Tay Ho	120 (100 - 139)	74.7 (62.4 - 86.3)	3,623.2 (2,942.0 - 4,315.0)	2,257.5 (1,833.1 - 2,688.5)	5.0 (4.0 - 6.1)
Long Bien	198 (166 - 229)	61.4 (51.4 - 71.0)	6,131.3 (4,983.3 - 7,295.3)	1,900.9 (1,545.0 - 2,261.8)	5.0 (4.0 - 6.0)
Hai Ba Trung	251 (210 - 290)	82.6 (69.1 - 95.5)	7,366.1 (5,975.6 - 8,780.5)	2,426.4 (1,968.3 - 2,892.3)	5.0 (4.0 - 6.0)
Hoan Kiem	130 (108 - 150)	95.5 (79.8 - 110.5)	3,728.8 (3,022.8 - 4,447.7)	2,749.5 (2,228.9 - 3,279.6)	5.0 (4.0 - 6.0)
Me Linh	147 (123 - 170)	61.1 (51.1 - 70.7)	4,367.4 (3,545.2 - 5,202.8)	1,815.6 (1,473.8 - 2,162.8)	4.9 (4.0 - 5.9)
Ung Hoa	173 (144 - 200)	81.9 (68.4 - 94.7)	4,939.4 (4,004.0 - 5,891.9)	2,342.4 (1,898.8 - 2,794.1)	4.9 (3.9 - 5.9)
Chuong My	219 (183 - 253)	64.9 (54.2 - 75.1)	6,489.3 (5,267.4 - 7,730.8)	1,923.7 (1,561.5 - 2,291.8)	4.9 (3.9 - 5.9)
Dong Da	295 (247 - 342)	79.4 (66.4 - 91.9)	8,591.7 (6,969.5 - 10,241.7)	2,312.1 (1,875.5 - 2,756.1)	4.9 (3.9 - 5.9)
Dong Anh	232 (194 - 268)	57.1 (47.7 - 66.0)	7,067.9 (5,744.0 - 8,409.9)	1,741.9 (1,415.7 - 2,072.7)	4.8 (3.9 - 5.8)
Dan Phuong	120 (100 - 139)	68.7 (57.4 - 79.5)	3,507.3 (2,846.4 - 4,178.8)	2,009.9 (1,631.2 - 2,394.7)	4.8 (3.9 - 5.8)
Phuc Tho	138 (116 - 160)	75.2 (62.8 - 87.1)	3,976.4 (3,225.1 - 4,740.7)	2,160.8 (1,752.5 - 2,576.1)	4.8 (3.8 - 5.8)
Ba Dinh	189 (158 - 219)	85.2 (71.2 - 98.6)	5,489.3 (4,453.9 - 6,541.8)	2,473.8 (2,007.2 - 2,948.2)	4.8 (3.8 - 5.8)
Phu Xuyen	165 (138 - 192)	77.3 (64.6 - 89.5)	4,779.0 (3,877.1 - 5,696.0)	2,233.3 (1,811.9 - 2,661.9)	4.8 (3.8 - 5.7)
Gia Lam	174 (145 - 201)	60.7 (50.7 - 70.3)	5,160.8 (4,192.1 - 6,143.5)	1,803.8 (1,465.2 - 2,147.3)	4.7 (3.8 - 5.6)

Soc Son	186 (156 - 216)	54.3 (45.3 - 62.9)	5,587.9 (4,542.3 - 6,647.1)	1,627.1 (1,322.6 - 1,935.5)	4.5 (3.6 - 5.4)
Quoc Oai	119 (99 - 138)	61.2 (51.1 - 71.0)	3,456.0 (2,807.1 - 4,114.0)	1,777.6 (1,443.9 - 2,116.1)	4.4 (3.5 - 5.3)
Son Tay	91 (76 - 106)	62.5 (52.1 - 72.5)	2,711.9 (2,205.0 - 3,225.0)	1,859.3 (1,511.8 - 2,211.1)	4.3 (3.5 - 5.2)
My Duc	126 (105 - 146)	62.8 (52.3 - 73.0)	3,507.6 (2,848.1 - 4,176.5)	1,754.7 (1,424.7 - 2,089.3)	4.1 (3.3 - 4.9)
Thach That	119 (99 - 138)	55.0 (45.8 - 63.8)	3,416.6 (2,777.1 - 4,063.9)	1,577.7 (1,282.4 - 1,876.6)	4.0 (3.2 - 4.8)
Ba Vi	178 (148 - 207)	61.3 (51.0 - 71.2)	5,058.3 (4,110.3 - 6,018.5)	1,740.8 (1,414.5 - 2,071.2)	4.0 (3.2 - 4.8)
Bac Ninh*	***946 (789 - 1,096)***	***69.1 (57.7 - 80.0)***	***23,779.8 (19,353.7 - 28,252.8)***	***1,737.2 (1,413.9 - 2,064.0)***	***3.8 (3.1 - 4.6)***
Yen Phong	110 (92 - 127)	56.9 (47.5 - 65.7)	2,892.9 (2,355.9 - 3,435.4)	1,501.5 (1,222.7 - 1,783.0)	4.2 (3.4 - 5.0)
Tu Son	123 (103 - 143)	70.0 (58.5 - 80.9)	3,163.3 (2,574.3 - 3,759.0)	1,793.2 (1,459.3 - 2,130.8)	4.1 (3.3 - 4.9)
Tien Du	118 (98 - 136)	66.6 (55.6 - 77.2)	2,927.3 (2,382.0 - 3,478.5)	1,658.9 (1,349.9 - 1,971.2)	3.8 (3.0 - 4.5)
Que Vo	126 (105 - 146)	64.3 (53.7 - 74.5)	3,177.9 (2,587.0 - 3,774.7)	1,624.1 (1,322.2 - 1,929.2)	3.8 (3.0 - 4.5)
Bac Ninh	142 (119 - 165)	57.5 (48.0 - 66.6)	3,730.1 (3,039.8 - 4,426.0)	1,505.9 (1,227.2 - 1,786.8)	3.8 (3.0 - 4.5)
Thuan Thanh	132 (110 - 153)	77.0 (64.2 - 89.3)	3,279.3 (2,668.5 - 3,896.8)	1,907.2 (1,552.0 - 2,266.3)	3.7 (3.0 - 4.4)
Gia Binh	94 (78 - 109)	90.8 (75.7 - 105.3)	2,238.9 (1,819.9 - 2,663.0)	2,162.8 (1,758.1 - 2,572.6)	3.7 (2.9 - 4.4)
Luong Tai	101 (84 - 117)	96.4 (80.3 - 111.8)	2,370.1 (1,926.3 - 2,819.4)	2,268.7 (1,843.9 - 2,698.8)	3.6 (2.9 - 4.3)
Hung Yen*	***658 (548 - 763)***	***52.5 (43.7 - 60.9)***	***23,940.0 (19,392.6 - 28,573.7)***	***1,911.0 (1,548.0 - 2,280.9)***	***5.7 (4.6 - 6.8)***

Khoai Chau	112 (94 - 130)	59.6 (49.7 - 69.0)	4,087.2 (3,307.3 - 4,883.6)	2,171.1 (1,756.8 - 2,594.1)	6.1 (4.9 - 7.3)
Van Giang	65 (54 - 75)	53.8 (44.9 - 62.3)	2,403.3 (1,946.4 - 2,869.3)	1,989.5 (1,611.3 - 2,375.2)	6.0 (4.8 - 7.2)
Yen My	70 (58 - 81)	44.8 (37.4 - 51.9)	2,606.8 (2,112.8 - 3,109.9)	1,667.5 (1,351.5 - 1,989.3)	5.8 (4.7 - 7.0)
My Hao	51 (42 - 59)	44.9 (37.4 - 52.0)	1,889.3 (1,531.4 - 2,253.6)	1,675.6 (1,358.2 - 1,998.7)	5.8 (4.7 - 7.0)
Van Lam	58 (48 - 67)	43.5 (36.3 - 50.4)	2,154.8 (1,746.8 - 2,570.0)	1,619.8 (1,313.1 - 1,932.0)	5.7 (4.6 - 6.9)
Kim Dong	69 (58 - 81)	59.0 (49.2 - 68.4)	2,492.7 (2,018.0 - 2,976.9)	2,117.3 (1,714.1 - 2,528.5)	5.7 (4.6 - 6.8)
An Thi	75 (63 - 87)	56.1 (46.7 - 65.1)	2,681.9 (2,172.0 - 3,201.6)	1,995.4 (1,616.0 - 2,382.1)	5.4 (4.4 - 6.5)
Hung Yen	57 (48 - 66)	49.2 (40.9 - 57.0)	2,105.4 (1,707.4 - 2,510.1)	1,809.5 (1,467.4 - 2,157.3)	5.4 (4.3 - 6.5)
Tien Lu	52 (43 - 61)	56.0 (46.6 - 65.1)	1,842.6 (1,492.7 - 2,198.7)	1,978.7 (1,603.1 - 2,361.2)	5.2 (4.2 - 6.3)
Phu Cu	48 (40 - 55)	59.6 (49.6 - 69.3)	1,676.0 (1,357.7 - 2,000.0)	2,096.2 (1,698.2 - 2,501.5)	5.2 (4.1 - 6.2)
Ha Nam*	***811 (675 - 942)***	***95.1 (79.1 - 110.4)***	***19,520.0 (15,881.1 - 23,196.9)***	***2,288.9 (1,862.2 - 2,720.1)***	***3.4 (2.8 - 4.1)***
Duy Tien	140 (117 - 163)	102.4 (85.4 - 118.6)	3,427.6 (2,786.4 - 4,076.8)	2,499.2 (2,031.7 - 2,972.5)	3.9 (3.1 - 4.7)
Thanh Liem	143 (119 - 165)	90.2 (75.2 - 104.5)	3,566.7 (2,902.7 - 4,237.6)	2,254.4 (1,834.7 - 2,678.4)	3.7 (3.0 - 4.5)
Ly Nhan	188 (157 - 219)	104.6 (87.0 - 121.5)	4,439.2 (3,610.2 - 5,277.4)	2,463.6 (2,003.5 - 2,928.8)	3.4 (2.7 - 4.0)
Binh Luc	137 (114 - 160)	103.2 (85.8 - 119.9)	3,233.1 (2,629.7 - 3,842.8)	2,430.0 (1,976.5 - 2,888.4)	3.3 (2.7 - 4.0)
Phu Ly	104 (86 - 121)	87.7 (72.8 - 102.0)	2,467.8 (2,008.9 - 2,930.7)	2,081.3 (1,694.3 - 2,471.7)	3.1 (2.5 - 3.7)

Kim Bang	98 (81 - 114)	78.0 (64.8 - 90.8)	2,385.6 (1,943.2 - 2,831.4)	1,898.9 (1,546.7 - 2,253.7)	3.0 (2.5 - 3.6)
Hai Duong*	***968 (805 - 1,124)***	***51.1 (42.5 - 59.4)***	***32,989.8 (26,753.7 - 39,327.5)***	***1,743.4 (1,413.9 - 2,078.3)***	***4.8 (3.9 - 5.8)***
Nam Sach	75 (63 - 87)	59.6 (49.6 - 69.1)	2,563.4 (2,076.0 - 3,060.3)	2,029.2 (1,643.4 - 2,422.5)	5.3 (4.3 - 6.4)
Hai Duong	111 (93 - 129)	46.0 (38.4 - 53.4)	3,991.2 (3,238.8 - 4,755.5)	1,653.6 (1,341.8 - 1,970.2)	5.3 (4.3 - 6.4)
Cam Giang	75 (62 - 87)	50.5 (42.0 - 58.5)	2,579.5 (2,090.4 - 3,077.5)	1,745.1 (1,414.2 - 2,082.1)	5.3 (4.3 - 6.4)
Binh Giang	61 (50 - 70)	50.8 (42.3 - 59.0)	2,071.1 (1,679.1 - 2,469.7)	1,737.1 (1,408.3 - 2,071.4)	5.0 (4.0 - 6.0)
Gia Loc	84 (70 - 97)	55.5 (46.2 - 64.4)	2,827.6 (2,291.5 - 3,373.3)	1,868.9 (1,514.5 - 2,229.6)	5.0 (4.0 - 5.9)
Thanh Mien	78 (65 - 91)	56.8 (47.2 - 65.9)	2,613.1 (2,117.6 - 3,117.3)	1,899.9 (1,539.6 - 2,266.5)	4.9 (3.9 - 5.9)
Kim Thanh	67 (56 - 78)	49.1 (40.8 - 57.1)	2,283.3 (1,852.5 - 2,720.9)	1,677.1 (1,360.6 - 1,998.4)	4.7 (3.8 - 5.6)
Ninh Giang	81 (67 - 94)	55.0 (45.7 - 64.0)	2,702.9 (2,191.8 - 3,222.3)	1,845.1 (1,496.2 - 2,199.6)	4.7 (3.7 - 5.6)
Thanh Ha	92 (77 - 108)	56.3 (46.8 - 65.4)	3,077.5 (2,495.1 - 3,669.6)	1,871.8 (1,517.5 - 2,231.9)	4.6 (3.7 - 5.6)
Kinh Mon	82 (68 - 95)	47.6 (39.5 - 55.3)	2,798.7 (2,271.6 - 3,333.5)	1,622.1 (1,316.6 - 1,932.0)	4.5 (3.6 - 5.4)
Tu Ky	93 (77 - 108)	52.3 (43.5 - 60.9)	3,091.1 (2,507.3 - 3,684.1)	1,744.4 (1,414.9 - 2,079.1)	4.5 (3.6 - 5.4)
Chi Linh	69 (58 - 81)	40.4 (33.5 - 47.0)	2,390.1 (1,942.2 - 2,843.5)	1,390.6 (1,130.0 - 1,654.4)	4.1 (3.3 - 5.0)
Thai Binh*	***1,738 (1,443 - 2,022)***	***93.4 (77.6 - 108.7)***	***39,772.4 (32,368.7 - 47,244.1)***	***2,137.8 (1,739.8 - 2,539.4)***	***2.9 (2.4 - 3.5)***
Hung Ha	257 (214 - 299)	101.5 (84.4 - 117.9)	5,955.6 (4,844.3 - 7,078.7)	2,351.5 (1,912.7 - 2,794.9)	3.3 (2.6 - 3.9)

Vu Thu	228 (190 - 266)	100.2 (83.3 - 116.5)	5,222.2 (4,248.0 - 6,206.4)	2,291.2 (1,863.8 - 2,723.0)	3.1 (2.5 - 3.7)
Thai Binh	165 (137 - 192)	80.2 (66.7 - 93.3)	3,997.3 (3,256.6 - 4,743.6)	1,940.1 (1,580.6 - 2,302.3)	3.1 (2.5 - 3.7)
Dong Hung	251 (208 - 292)	102.4 (85.0 - 119.1)	5,653.6 (4,598.8 - 6,719.2)	2,309.1 (1,878.3 - 2,744.3)	3.0 (2.4 - 3.6)
Quynh Phu	231 (192 - 269)	95.8 (79.6 - 111.5)	5,323.8 (4,332.9 - 6,323.8)	2,209.6 (1,798.3 - 2,624.6)	3.0 (2.4 - 3.6)
Kien Xuong	208 (172 - 242)	95.9 (79.6 - 111.7)	4,660.4 (3,792.6 - 5,536.1)	2,150.8 (1,750.3 - 2,554.9)	2.8 (2.2 - 3.3)
Thai Thuy	239 (198 - 278)	93.5 (77.5 - 109.1)	5,290.3 (4,305.7 - 6,283.4)	2,072.8 (1,687.1 - 2,461.9)	2.6 (2.1 - 3.1)
Tien Hai	159 (132 - 185)	73.7 (61.1 - 86.0)	3,669.2 (2,989.7 - 4,353.1)	1,702.4 (1,387.1 - 2,019.7)	2.5 (2.0 - 3.0)
Hai Phong*	***1,661 (1,380 - 1,933)***	***81.9 (68.0 - 95.3)***	***38,360.9 (31,294.0 - 45,461.7)***	***1,891.7 (1,543.2 - 2,241.8)***	***2.8 (2.2 - 3.3)***
Ngo Quyen	155 (129 - 180)	94.0 (78.3 - 109.2)	3,685.9 (3,006.8 - 4,368.5)	2,229.7 (1,818.9 - 2,642.6)	3.1 (2.5 - 3.7)
Hong Bang	89 (74 - 103)	92.5 (77.0 - 107.4)	2,109.6 (1,721.1 - 2,500.0)	2,194.9 (1,790.7 - 2,601.2)	3.1 (2.5 - 3.7)
Hai An	95 (79 - 111)	71.7 (59.6 - 83.3)	2,347.0 (1,916.8 - 2,778.6)	1,765.4 (1,441.8 - 2,090.1)	2.9 (2.4 - 3.5)
Le Chan	195 (163 - 227)	89.0 (74.0 - 103.4)	4,580.6 (3,736.9 - 5,428.4)	2,084.3 (1,700.4 - 2,470.1)	2.9 (2.4 - 3.5)
An Duong	147 (122 - 171)	75.1 (62.4 - 87.3)	3,491.7 (2,849.6 - 4,136.5)	1,784.1 (1,456.0 - 2,113.5)	2.9 (2.3 - 3.5)
Thuy Nguyen	268 (223 - 312)	80.3 (66.7 - 93.4)	6,145.6 (5,012.2 - 7,284.9)	1,841.0 (1,501.5 - 2,182.4)	2.8 (2.3 - 3.4)
An Lao	125 (104 - 145)	85.1 (70.6 - 99.1)	2,779.7 (2,266.1 - 3,296.4)	1,894.7 (1,544.6 - 2,246.8)	2.7 (2.2 - 3.2)
Vinh Bao	181 (150 - 211)	98.9 (82.1 - 115.2)	3,942.9 (3,213.4 - 4,676.9)	2,156.5 (1,757.6 - 2,558.0)	2.6 (2.1 - 3.1)

Kien An	72 (60 - 84)	61.3 (50.9 - 71.4)	1,793.0 (1,465.1 - 2,121.6)	1,518.9 (1,241.1 - 1,797.2)	2.6 (2.1 - 3.1)
Duong Kinh	41 (34 - 48)	68.1 (56.5 - 79.3)	965.4 (788.1 - 1,143.4)	1,600.5 (1,306.5 - 1,895.6)	2.6 (2.1 - 3.1)
Tien Lang	130 (108 - 151)	83.9 (69.6 - 97.8)	2,885.2 (2,352.9 - 3,420.2)	1,864.0 (1,520.1 - 2,209.6)	2.5 (2.0 - 3.0)
Kien Thuy	108 (90 - 126)	77.2 (64.0 - 90.0)	2,408.5 (1,964.4 - 2,854.9)	1,715.3 (1,398.9 - 2,033.1)	2.5 (2.0 - 2.9)
Do Son	34 (28 - 40)	69.7 (57.7 - 81.3)	789.8 (644.8 - 935.2)	1,610.8 (1,315.1 - 1,907.4)	2.3 (1.9 - 2.7)
Cat Hai	19 (16 - 23)	60.3 (49.9 - 70.6)	436.0 (356.0 - 516.2)	1,358.7 (1,109.4 - 1,608.5)	1.8 (1.5 - 2.2)
Ninh Binh*	***758 (628 - 883)***	***77.1 (64.0 - 89.8)***	***18,422.6 (15,029.6 - 21,830.2)***	***1,875.1 (1,529.8 - 2,221.9)***	***2.7 (2.2 - 3.2)***
Ninh Binh	102 (85 - 119)	79.5 (66.1 - 92.4)	2,626.6 (2,144.3 - 3,110.5)	2,044.3 (1,669.0 - 2,421.0)	3.0 (2.4 - 3.6)
Gia Vien	111 (92 - 129)	91.8 (76.2 - 106.9)	2,623.2 (2,138.1 - 3,111.4)	2,168.1 (1,767.2 - 2,571.6)	2.9 (2.3 - 3.4)
Yen Khanh	126 (104 - 146)	85.4 (70.9 - 99.5)	2,988.8 (2,436.8 - 3,544.0)	2,032.3 (1,656.9 - 2,409.8)	2.8 (2.3 - 3.4)
Hoa Lu	65 (54 - 75)	90.1 (74.7 - 105.0)	1,495.3 (1,218.7 - 1,773.6)	2,081.5 (1,696.5 - 2,468.9)	2.7 (2.2 - 3.2)
Kim Son	114 (95 - 133)	62.3 (51.7 - 72.6)	2,927.2 (2,390.8 - 3,464.6)	1,600.1 (1,306.9 - 1,893.8)	2.6 (2.1 - 3.1)
Yen Mo	98 (81 - 115)	82.9 (68.7 - 96.7)	2,286.6 (1,864.3 - 2,711.2)	1,930.1 (1,573.6 - 2,288.6)	2.6 (2.1 - 3.1)
Nho Quan	107 (88 - 125)	71.3 (59.0 - 83.2)	2,574.6 (2,101.0 - 3,049.8)	1,718.3 (1,402.3 - 2,035.5)	2.4 (2.0 - 2.9)
Tam Diep	35 (29 - 41)	55.5 (46.0 - 64.9)	900.2 (735.6 - 1,064.9)	1,431.9 (1,170.1 - 1,694.0)	2.2 (1.8 - 2.6)
Ho Chi Minh*	***4,076 (3,377 - 4,754)***	***45.3 (37.5 - 52.9)***	***88,710.3 (72,525.0 - 104,897.0)***	***986.4 (806.5 - 1,166.4)***	***1.9 (1.6 - 2.3)***

District 10	178 (147 - 207)	75.6 (62.7 - 88.1)	3,531.5 (2,882.2 - 4,183.2)	1,503.9 (1,227.4 - 1,781.5)	2.1 (1.7 - 2.6)
District 11	145 (120 - 169)	69.0 (57.3 - 80.5)	3,004.5 (2,453.9 - 3,556.3)	1,431.6 (1,169.3 - 1,694.5)	2.1 (1.7 - 2.6)
Tan Binh	256 (212 - 298)	53.9 (44.7 - 62.9)	5,366.9 (4,384.5 - 6,350.9)	1,130.4 (923.5 - 1,337.6)	2.1 (1.7 - 2.5)
Tan Phu	199 (165 - 232)	41.1 (34.1 - 47.8)	4,583.0 (3,750.0 - 5,414.8)	944.3 (772.6 - 1,115.7)	2.1 (1.7 - 2.5)
District 3	166 (138 - 194)	87.4 (72.5 - 101.9)	3,202.2 (2,611.8 - 3,795.3)	1,682.1 (1,371.9 - 1,993.6)	2.1 (1.7 - 2.5)
Phu Nhuan	135 (112 - 158)	82.6 (68.4 - 96.3)	2,616.0 (2,133.9 - 3,100.2)	1,595.5 (1,301.5 - 1,890.8)	2.1 (1.7 - 2.5)
District 5	125 (103 - 146)	78.5 (65.1 - 91.5)	2,475.9 (2,020.5 - 2,932.8)	1,556.4 (1,270.2 - 1,843.7)	2.1 (1.7 - 2.5)
District 1	121 (100 - 141)	84.6 (70.1 - 98.6)	2,344.6 (1,912.8 - 2,778.2)	1,643.9 (1,341.1 - 1,947.9)	2.1 (1.7 - 2.5)
District 4	114 (94 - 132)	64.8 (53.7 - 75.6)	2,386.9 (1,950.1 - 2,824.3)	1,361.4 (1,112.2 - 1,610.8)	2.1 (1.7 - 2.5)
District 6	150 (124 - 175)	64.3 (53.3 - 74.9)	3,156.8 (2,579.2 - 3,735.2)	1,351.6 (1,104.3 - 1,599.3)	2.1 (1.7 - 2.5)
Binh Thanh	309 (256 - 360)	61.9 (51.3 - 72.1)	6,332.0 (5,171.3 - 7,495.1)	1,268.5 (1,036.0 - 1,501.5)	2.1 (1.7 - 2.5)
Go Vap	288 (239 - 336)	42.6 (35.3 - 49.7)	6,426.1 (5,255.7 - 7,595.8)	949.3 (776.4 - 1,122.1)	2.0 (1.6 - 2.4)
District 12	204 (169 - 238)	32.9 (27.2 - 38.3)	4,879.8 (3,995.5 - 5,761.7)	786.9 (644.3 - 929.1)	2.0 (1.6 - 2.3)
Binh Tan	235 (195 - 274)	30.0 (24.9 - 35.0)	5,915.4 (4,846.8 - 6,979.6)	754.3 (618.1 - 890.1)	1.9 (1.6 - 2.3)
District 8	225 (186 - 262)	52.9 (43.9 - 61.8)	4,856.7 (3,970.4 - 5,743.2)	1,143.7 (934.9 - 1,352.4)	1.9 (1.6 - 2.3)
Thu Duc	196 (163 - 229)	33.1 (27.4 - 38.6)	4,516.4 (3,695.9 - 5,335.5)	762.0 (623.6 - 900.2)	1.9 (1.6 - 2.3)

District 2	79 (66 - 92)	43.9 (36.3 - 51.2)	1,736.2 (1,419.8 - 2,052.5)	963.1 (787.6 - 1,138.6)	1.9 (1.5 - 2.3)
District 7	127 (106 - 149)	35.4 (29.3 - 41.3)	2,940.1 (2,406.0 - 3,473.2)	816.3 (668.1 - 964.4)	1.9 (1.5 - 2.2)
District 9	121 (100 - 141)	30.5 (25.2 - 35.6)	2,765.1 (2,262.6 - 3,266.7)	696.5 (569.9 - 822.8)	1.8 (1.4 - 2.1)
Hoc Mon	198 (164 - 232)	36.5 (30.2 - 42.7)	4,462.6 (3,650.9 - 5,273.2)	823.0 (673.3 - 972.5)	1.8 (1.4 - 2.1)
Binh Chanh	217 (179 - 253)	30.7 (25.4 - 35.9)	5,121.7 (4,192.7 - 6,048.2)	726.0 (594.3 - 857.3)	1.7 (1.4 - 2.0)
Nha Be	66 (54 - 77)	31.7 (26.2 - 37.1)	1,504.1 (1,230.8 - 1,776.8)	727.2 (595.0 - 859.0)	1.6 (1.3 - 1.9)
Cu Chi	194 (160 - 227)	42.0 (34.7 - 49.1)	4,024.9 (3,289.4 - 4,760.7)	871.1 (711.9 - 1,030.4)	1.6 (1.3 - 1.9)
Can Gio	27 (23 - 32)	38.3 (31.6 - 44.8)	560.9 (458.4 - 663.4)	784.2 (640.9 - 927.5)	1.4 (1.1 - 1.6)
Quang Ninh*	***687 (569 - 801)***	***52.0 (43.1 - 60.7)***	***18,436.3 (15,042.3 - 21,844.0)***	***1,396.3 (1,139.3 - 1,654.4)***	***2.5 (2.1 - 3.0)***
Quang Yen	107 (89 - 125)	73.6 (61.1 - 85.6)	2,775.0 (2,260.1 - 3,294.1)	1,901.8 (1,548.9 - 2,257.4)	3.2 (2.6 - 3.8)
Dong Trieu	111 (92 - 129)	64.6 (53.6 - 75.4)	2,884.3 (2,351.7 - 3,419.9)	1,680.1 (1,369.8 - 1,992.1)	2.7 (2.2 - 3.2)
Mong Cai	47 (39 - 55)	43.5 (36.1 - 50.8)	1,312.7 (1,071.8 - 1,554.3)	1,209.3 (987.4 - 1,431.9)	2.6 (2.1 - 3.1)
Ha Long	145 (120 - 169)	53.7 (44.5 - 62.7)	3,973.0 (3,243.0 - 4,705.5)	1,471.2 (1,200.9 - 1,742.4)	2.6 (2.1 - 3.1)
Uong Bi	63 (52 - 73)	51.9 (43.0 - 60.6)	1,705.5 (1,392.0 - 2,020.2)	1,409.7 (1,150.6 - 1,669.8)	2.6 (2.1 - 3.1)
Co To	2 (2 - 2)	29.5 (24.5 - 34.5)	57.2 (46.8 - 67.7)	910.8 (745.1 - 1,076.5)	2.4 (2.0 - 2.9)
Tien Yen	22 (18 - 26)	43.1 (35.7 - 50.4)	578.1 (471.7 - 684.8)	1,137.3 (928.0 - 1,347.3)	2.4 (1.9 - 2.8)

Cam Pha	89 (74 - 104)	47.0 (38.9 - 54.9)	2,491.2 (2,034.6 - 2,948.6)	1,309.5 (1,069.6 - 1,550.0)	2.3 (1.9 - 2.8)
Van Don	23 (19 - 26)	48.4 (40.0 - 56.6)	591.4 (482.5 - 700.6)	1,268.6 (1,035.2 - 1,502.9)	2.3 (1.9 - 2.7)
Dam Ha	17 (14 - 20)	42.0 (34.8 - 49.1)	453.3 (369.8 - 537.0)	1,099.7 (897.3 - 1,302.8)	2.3 (1.8 - 2.7)
Hai Ha	24 (20 - 28)	39.0 (32.2 - 45.6)	642.9 (524.8 - 761.3)	1,044.2 (852.4 - 1,236.5)	2.2 (1.8 - 2.7)
Hoanh Bo	20 (16 - 23)	37.4 (30.9 - 43.7)	527.3 (430.5 - 624.3)	1,001.3 (817.6 - 1,185.5)	2.1 (1.7 - 2.5)
Ba Che	7 (6 - 8)	30.9 (25.5 - 36.1)	184.7 (150.9 - 218.7)	835.8 (682.6 - 989.3)	2.0 (1.6 - 2.4)
Binh Lieu	10 (8 - 11)	30.7 (25.3 - 35.9)	259.6 (212.0 - 307.3)	820.7 (670.2 - 971.4)	1.9 (1.5 - 2.2)
Dien Bien*	***222 (183 - 259)***	***37.0 (30.6 - 43.3)***	***6,437.5 (5,258.7 - 7,617.9)***	***1,075.0 (878.1 - 1,272.1)***	***2.2 (1.8 - 2.6)***
Dien Bien Phu	34 (28 - 40)	57.9 (47.9 - 67.7)	965.0 (787.9 - 1,142.4)	1,649.0 (1,346.4 - 1,952.2)	2.3 (1.9 - 2.8)
Muong Lay	7 (5 - 8)	58.6 (48.5 - 68.5)	181.2 (147.9 - 214.6)	1,623.6 (1,325.2 - 1,922.8)	2.3 (1.9 - 2.7)
Tua Chua	18 (15 - 21)	30.7 (25.4 - 35.9)	521.3 (425.9 - 616.8)	907.2 (741.3 - 1,073.4)	2.2 (1.8 - 2.6)
Muong Nhe	10 (8 - 12)	22.3 (18.5 - 26.1)	331.1 (270.9 - 391.3)	724.1 (592.4 - 855.8)	2.2 (1.8 - 2.6)
Nam Po	13 (11 - 16)	24.2 (20.0 - 28.3)	414.9 (339.3 - 490.6)	755.7 (617.9 - 893.4)	2.2 (1.8 - 2.6)
Muong Cha	14 (11 - 16)	28.4 (23.5 - 33.2)	409.6 (334.8 - 484.5)	853.3 (697.4 - 1,009.3)	2.2 (1.8 - 2.6)
Muong Ang	18 (15 - 21)	37.3 (30.8 - 43.6)	518.8 (423.8 - 614.0)	1,071.6 (875.3 - 1,268.3)	2.2 (1.7 - 2.6)
Tuan Giao	33 (27 - 39)	37.8 (31.2 - 44.2)	950.3 (776.1 - 1,124.7)	1,081.3 (883.2 - 1,279.8)	2.1 (1.7 - 2.5)

Dien Bien	56 (47 - 66)	47.1 (38.9 - 55.1)	1,581.3 (1,291.2 - 1,872.0)	1,321.1 (1,078.7 - 1,563.9)	2.1 (1.7 - 2.5)
Dien Bien Dong	19 (15 - 22)	27.9 (23.1 - 32.7)	563.9 (460.9 - 667.0)	840.6 (687.1 - 994.3)	2.1 (1.7 - 2.5)

*the provincial name.
